# Dietary phytoestrogen intake and ovarian cancer risk: a prospective study in the prostate, lung, colorectal and ovarian (PLCO) cohort

**DOI:** 10.1093/carcin/bgae015

**Published:** 2024-02-20

**Authors:** Yizuo Song, Huijun Huang, Mingmin Jin, Binwei Cheng, Shanshan Wang, Xinjun Yang, Xiaoli Hu

**Affiliations:** Department of Obstetrics and Gynecology, The First Affiliated Hospital of Wenzhou Medical University, Wenzhou 325000, Zhejiang, China; Zhejiang Provincial Clinical Research Center for Obstetrics and Gynecology, The First Affiliated Hospital of Wenzhou Medical University, Wenzhou 325000, Zhejiang, China; Department of Epidemiology and Health Statistics, School of Public Health and Management, Wenzhou Medical University, Wenzhou 325000, Zhejiang, China; Department of Epidemiology and Health Statistics, School of Public Health and Management, Wenzhou Medical University, Wenzhou 325000, Zhejiang, China; Department of Epidemiology and Health Statistics, School of Public Health and Management, Wenzhou Medical University, Wenzhou 325000, Zhejiang, China; Department of Epidemiology and Health Statistics, School of Public Health and Management, Wenzhou Medical University, Wenzhou 325000, Zhejiang, China; Department of Epidemiology and Health Statistics, School of Public Health and Management, Wenzhou Medical University, Wenzhou 325000, Zhejiang, China; Department of Obstetrics and Gynecology, The First Affiliated Hospital of Wenzhou Medical University, Wenzhou 325000, Zhejiang, China; Zhejiang Provincial Clinical Research Center for Obstetrics and Gynecology, The First Affiliated Hospital of Wenzhou Medical University, Wenzhou 325000, Zhejiang, China

## Abstract

Estrogen plays a crucial role in ovarian tumorigenesis. Phytoestrogens (PEs) are a type of daily dietary nutrient for humans and possess a mild estrogenic characteristic. This study aimed to assess the correlation of the consumption of dietary PEs with ovarian cancer risk using data in the prostate, lung, colorectal and ovarian (PLCO) cancer screening trial. Participants were enrolled in PLCO from 1993 to 2001. Hazard ratios (HR) and 95% confidence intervals (CI) were utilized to determine the association between the intake of PEs and ovarian cancer occurrence, which were calculated by the Cox proportional hazards regression analysis. In total, 24 875 participants were identified upon completion of the initial dietary questionnaire (DQX). Furthermore, the analysis also included a total of 45 472 women who filled out the diet history questionnaire (DHQ). Overall, after adjustment for confounders, the dietary intake of total PEs was significantly associated with the risk of ovarian cancer in the DHQ group (HR_*Q*4vs*Q*1_ = 0.69, 95% CI: 0.50–0.95; *P* for trend = 0.066). Especially, individuals who consumed the highest quartile of isoflavones were found to have a decreased risk of ovarian cancer in the DHQ group (HR_*Q*4vs*_Q_*1_ = 0.68, 95% CI: 0.50–0.94; *P* for trend = 0.032). However, no such significant associations were observed for the DQX group. In summary, this study suggests that increased dietary intake of total PEs especially isoflavones was linked with a lower risk for developing ovarian cancer. More research is necessary to validate the findings and explore the potential mechanisms.

## Teaser

We evaluate the role of phytoestrogens in ovarian cancer risk based on the data from PLCO. Increased dietary intake of total PEs especially isoflavones was linked with a lower risk for developing ovarian cancer.

## Graphical Abstract

**Graphical Abstract:**
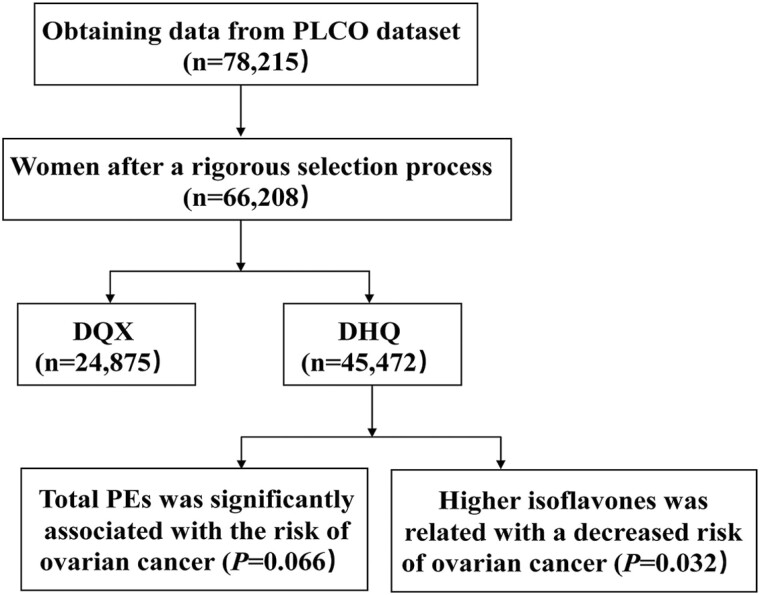

## Introduction

In 2020, ~207 252 women worldwide succumbed to ovarian cancer, making it still the deadliest gynecologic cancer (1). Due to the lack of efficient methods for early screening and identification, ovarian cancer is frequently detected in its advanced stages, resulting in an unfavorable prognosis (2). The etiology of ovarian cancer is still unknown, although the established risk factors include genetic mutation, menopausal hormone use, obesity and smoking cigarettes (3). Thus, it is essential to identify modifiable risk factors for developing effective strategies in ovarian cancer prevention.

17β-Estradiol (also known as estrogen/E2) is a major estrogen synthesized and secreted by the ovaries (4). Women typically suffer the menopause in their 40s or 50s due to a permanent loss of hormone synthesis functions in ovaries, causing several post-menopausal symptoms including osteoporosis and hot flushes (5). The treatment of these unpleasant symptoms, particularly osteoporosis, is mostly alleviated by estrogen replacement therapy (5,6). Nevertheless, women under prolonged exposure to estrogen are more prone to develop ovarian cancer (7).

Phytoestrogens (PEs), which are a group of compounds obtained from soybean, have a structural resemblance to mammalian 17β-estradiol and imitate weak estrogenic activities (8). It is worth mentioning that soybean and its derivatives have been extensively consumed by humans due to their health benefits on cholesterol reduction and mitigation of cardiovascular disease risks (9). Hence, a better description of PEs and ovarian cancer risk will help to modify health behaviors and develop cancer prevention. Several studies have reported the potential anticancer functions of PEs on ovarian cancer cells based on mainly cellular experiments (10–13). However, the function of dietary PE intake on ovarian cancer occurrence remains unclear.

We therefore, performed the current study to evaluate the association between the dietary consumption of PEs and the risk of ovarian cancer in the prostate, lung, colorectal and ovarian (PLCO) cancer screening trial.

## Materials and methods

### Subjects and study design

**Figure 1.**
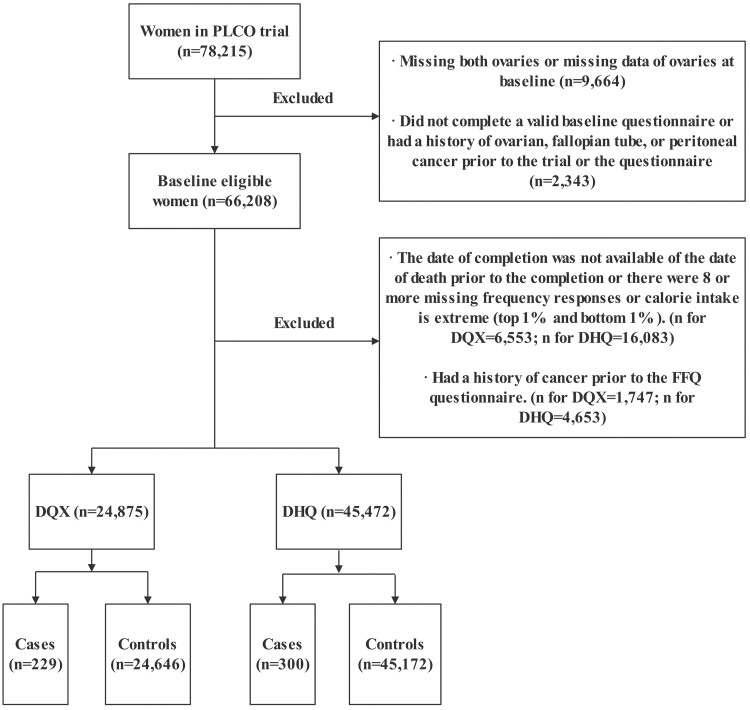
Flowchart for study population.

The PLCO study is a large prospective multi-center cancer screening trial designed to evaluate the effectiveness of selected screening modalities in reducing cancer-specific mortality from PLCO cancer (14). In brief, participants in the PLCO study were recruited via 10 centers in the US between 1993 and 2001. We restricted the present analysis to women (*n* = 78 215) because the outcome of interest was ovarian cancer. Subjects were eligible for inclusion who completed the baseline dietary questionnaire (DQX) and had no history of cancer prior to completing the diet history questionnaire (DHQ). In our analysis, women who were missing both ovaries or missing data of ovaries at baseline (*n* = 9664), did not complete a valid questionnaire at baseline or had a cancer history of ovarian, fallopian tube, or peritoneal before the trial or survey (*n* = 2343) were excluded. We then sequentially excluded 29 036 women from our analysis for the following reasons: unavailability of questionnaire completion date or death occurring before the completion or 8 or more missing frequency responses or extreme calorie intake (top 1% and bottom 1%) (*n* for DQX = 6553; *n* for DHQ = 16 083); a cancer history before the food-frequency questionnaire (FFQ) (*n* for DQX = 1747; *n* for DHQ = 4653). Following the aforementioned exclusions, a total of 45 472 women were included in this study (Figure 1). Study subjects were then defined as two groups, termed the DQX (*n* = 24 875) and the DHQ (*n* = 45 472).

### Data collection

Participants in both arms of the trial were offered a baseline questionnaire that gathered information on demographics (age, race, weight, height and education), smoking, family history of ovarian cancer, alcohol use, parity, duration of oral contraceptive and female hormones use. Dietary data were collected using two FFQs (DQX and DHQ), which included 137 (DQX) and 124 (DHQ) food items commonly consumed during the previous year in the United States, respectively. The detailed analysis output of the DietCalc software was used to calculate the quantity of nutrient intake. Nutrition data were primarily collected from the United States Department of Agriculture database and Agriculture’s 1994–96 Continuing Survey of Food Intakes. The total intake of PEs was determined by adding together the total amounts of isoflavones and coumestrol. Total isoflavone intake was defined as the sum of genistein, daidzein, glycitein, biochanin A and formononetin. The FFQ did not estimate the consumption of dietary lignan due to the unavailability of lignan amount in the database.

The baseline characteristics of the study population were determined based on the quartile of total daily PE intake. The quartiles for DHQ were as follows: first quartile (<250 μg/day), second quartile (250–470 μg/day), third quartile (470–930 μg/day), and fourth quartile (≥930 μg/day). The quartiles for DQX were as follows: first quartile (<237 μg/day), second quartile (237–437 μg/day), third quartile (437–788 μg/day) and fourth quartile (≥788 μg/day).

### Ascertainment of ovarian cancer

The study participants were given annual study update questionnaires to ascertain cancer cases. A medical record abstract review was used to collect cancer diagnosis information. The primary outcome for this study was incident primary ovarian, peritoneal or fallopian tube cancers, henceforth jointly referred to as ovarian cancer. The death status that occurred during the trial was acquired through the use of the annual study updates, reports from relatives, friends or physicians, and searches from the National Death Index.

### Statistical analysis

The data of DQX and DHQ groups were analyzed separately. Initially, the normality test was performed to assess the distribution of continuous variables. Chi-square tests were used to compare the demographic, anthropometric and lifestyle characteristics of the study populations across the quartile of total PE intake and case status for categorical variables, whereas ANOVA was employed for continuous variables. Descriptive statistics were utilized to report the uppermost and lowermost quartiles.

Cox proportional hazards regression model was employed to calculate hazard ratios (HR) and 95% confidence intervals (CI) for the upper quartile of PEs in comparison to the lowest quartile, which served as the reference group. The models were adjusted for variables that could potentially affect the results, including DHQ/DQX analysis entry age (continuous), body mass index (BMI) category (<18.5, 18.5–25, 25–30, >30 kg/m^2^, missing), ovarian cancer in the family history (yes, no and missing), duration of oral contraceptive usage (not applicable, ≤5, >5 years and missing) and years of female hormones use (not applicable, ≤5, >5 years and missing). In the multi-adjusted Cox proportional hazard model, a linear trend test was performed to evaluate the risk of ovarian cancer across all PEs. This test included each subtype of PEs (total PEs, total isoflavones and coumestrol) as a continuous variable. To explore a potential non-linear dose–response relationship between PEs and ovarian cancer risk, we performed restricted cubic spline models with four knots (fifth, 35th, 65th and 95th percentiles) of PEs in DHQ. Log transformation was used because of the positive skew of PEs intake. Stratified analyses were performed based on BMI (<25 and ≥25 kg/m^2^) and age (<65 and ≥65 years) in DHQ. The interactions between risk factors and phytoestrogens were assessed using the likelihood ratio test by comparing models with and without cross-product terms in the Cox proportional hazards regression models. All statistical analyses were conducted using the R 4.2.2. A *P* value < 0.05 (two-sided) was defined as statistically significant.

## Results

In the DQX sample set, 229 ovarian cancer cases were diagnosed among 24 875 women included in the final analysis during a median follow-up of 11.5 years. From the DHQ set, there was a total of 45 472 subjects with 300 ovarian cancer cases identified after a median of 8.9 years of follow-up. The demographic features of study subjects for DHQ and DQX were displayed in Tables 1 and 2, respectively, based on the distribution of quartile (*Q*_1_–*Q*_4_) of daily PE consumption. The average age of the participants was 65.37 years in DHQ and 62.58 years in DQX. Individuals who consumed higher amounts of PEs from both the DHQ and DQX were less likely to identify as non-Hispanic white, had a BMI ranging from 18.5 to 25, achieved a college or post-graduate education, were more likely to be never smokers, had less alcohol use and were more prone to have ≥1 live birth. Among individuals who finished the DHQ, there was no significant disparity in the years of oral contraceptive use; however, among participants in the DQX, a higher percentage in the *Q*_4_ of PEs had a longer history of oral contraceptive use. Reports indicated that individuals in the *Q*_4_ of PEs in both the DQX and DHQ experienced a longer female hormone use. In addition, participants with a greater consumption of PEs were also found to have a higher overall intake of energy compared with those in the *Q*_1_ group. No statistically significant difference was observed regarding family history of ovarian cancer in both DQX and DHQ.

**Table 1.** Baseline characteristics among quartile distribution of total phytoestrogen intake in DHQ

Characteristics	Total phytoestrogens (μg/day)					*P*-value1
	Total	First quartile (<250 μg/day)	Second quartile (250–470 μg/day)	Third quartile (470–930 μg/day)	Fourth quartile (≥930 μg/day)	
	*N* = 45 472	*N* = 11 111	*N* = 11 391	*N* = 11 522	*N* = 11 448	
**DHQ analysis entry age [years, mean (SD)]**	65.37 (5.75)	65.58 (5.81)	65.33 (5.68)	65.48 (5.72)	65.09 (5.78)	<0.001
**Study arm (%)**						0.111
Intervention	22 731 (49.99)	5461 (49.15)	5674 (49.81)	5843 (50.71)	5753 (50.25)	
Control	22 741 (50.01)	5650 (50.85)	5717 (50.19)	5679 (49.29)	5695 (49.75)	
**Race/ethnicity (%)**						<0.001
Non-Hispanic white	41 481 (91.22)	10 375 (93.38)	10 724 (94.14)	10 737 (93.19)	9645 (84.25)	
Non-Hispanic black	1717 (3.78)	547 (4.92)	429 (3.77)	356 (3.09)	385 (3.36)	
Asian	1488 (3.27)	41 (0.37)	83 (0.73)	236 (2.05)	1128 (9.85)	
Other	777 (1.71)	145 (1.31)	154 (1.35)	191 (1.66)	287 (2.51)	
Missing	9 (0.02)	3 (0.03)	1 (0.01)	2 (0.02)	3 (0.03)	
**BMI [kg/m** ^**2**^ **, mean (SD)]**	26.90 (5.40)	27.11 (5.48)	27.09 (5.41)	27 (5.34)	26.42 (5.34)	<0.001
**BMI category (%)**						<0.001
<18.5	482 (1.06)	105 (0.95)	103 (0.90)	119 (1.03)	155 (1.35)	
18.5–25	18 573 (40.84)	4443 (39.99)	4497 (39.48)	4518 (39.21)	5115 (44.68)	
25–30	15 452 (33.98)	3745 (33.71)	3907 (34.30)	4079 (35.40)	3721 (32.50)	
>30	10 415 (22.90)	2678 (24.10)	2729 (23.96)	2693 (23.37)	2315 (20.22)	
Missing	550 (1.21)	140 (1.26)	155 (1.36)	113 (0.98)	142 (1.24)	
**Education (%)**						<0.001
High school or less	14 883 (32.73)	4421 (39.79)	3927 (34.47)	3499 (30.37)	3036 (26.52)	
Post-high school training or some college	16 241 (35.72)	3923 (35.31)	4099 (35.98)	4151 (36.03)	4068 (35.53)	
College graduate or post-graduate	14 267 (31.38)	2745 (24.71)	3344 (29.36)	3853 (33.44)	4325 (37.78)	
Missing	81 (0.18)	22 (0.20)	21 (0.18)	19 (0.16)	19 (0.17)	
**Smoking status (%)**						<0.001
Never smoker	25 858 (56.87)	6022 (54.20)	6443 (56.56)	6720 (58.32)	6673 (58.29)	
Former smoker	3922 (8.63)	1267 (11.40)	1028 (9.02)	826 (7.17)	801 (7)	
Current smoker	15 689 (34.50)	3821 (34.39)	3919 (34.40)	3976 (34.51)	3973 (34.70)	
Missing	3 (0.01)	1 (0.01)	1 (0.01)	0 (0)	1 (0.01)	
**Family history of ovarian cancer (%)**						0.666
No	42 986 (94.53)	10 503 (94.53)	10 741 (94.29)	10 906 (94.65)	10 836 (94.65)	
Yes	1712 (3.76)	405 (3.65)	454 (3.99)	424 (3.68)	429 (3.75)	
Missing	774 (1.70)	203 (1.83)	196 (1.72)	192 (1.67)	183 (1.60)	
**Alcohol use (%)**						<0.001
No	13 672 (30.07)	3465 (31.19)	3167 (27.80)	3316 (28.78)	3724 (32.53)	
Yes	31 800 (69.93)	7646 (68.81)	8224 (72.20)	8206 (71.22)	7724 (67.47)	
**Parity (%)**						<0.001
No live births	4007 (8.81)	954 (8.59)	918 (8.06)	996 (8.64)	1139 (9.95)	
≥1 live birth	41 401 (91.05)	10 136 (91.22)	10 462 (91.84)	10 512 (91.23)	10 291 (89.89)	
Missing	64 (0.14)	21 (0.19)	11 (0.10)	14 (0.12)	18 (0.16)	
**Years of oral contraceptive use (%)**						0.047
Not applicable	20 296 (44.63)	5009 (45.08)	5034 (44.19)	5148 (44.68)	5105 (44.59)	
≤5	15 102 (33.21)	3626 (32.63)	3720 (32.66)	3848 (33.40)	3908 (34.14)	
>5	9981 (21.95)	2454 (22.09)	2613 (22.94)	2506 (21.75)	2408 (21.03)	
Missing	93 (0.20)	22 (0.20)	24 (0.21)	20 (0.17)	27 (0.24)	
**Years of female hormones use (%)**						<0.001
Not applicable	15 637 (34.39)	4227 (38.04)	3945 (34.63)	3815 (33.11)	3650 (31.88)	
≤5	14 518 (31.93)	3363 (30.27)	3654 (32.08)	3777 (32.78)	3724 (32.53)	
>5	14 982 (32.95)	3443 (30.99)	3715 (32.61)	3857 (33.48)	3967 (34.65)	
Missing	335 (0.74)	78 (0.70)	77 (0.68)	73 (0.63)	107 (0.93)	
**Total energy intake [kcal/day, median (IQR]**	1417.34 (1102.22, 1802.21)	1177.99 (915.47, 1489.62)	1388.48 (1,104.30, 1731.55)	1517.39 (1,200.28, 1898.44)	1592.09 (1,259.51, 2023.64)	<0.001
**Ovarian cancer (%)**						0.201
No	45 172 (99.34)	11 026 (99.23)	11 322 (99.39)	11 440 (99.29)	11 384 (99.44)	
Yes	300 (0.66)	85 (0.77)	69 (0.61)	82 (0.71)	64 (0.56)	

**Table 2.** Baseline characteristics among quartile distribution of total phytoestrogen intake in DQX

Characteristics	Total phytoestrogens (μg/day)					*P*-value1
	Total	First quartile (<237 μg/day)	Second quartile (237–437 μg/day)	Third quartile (437–788 μg/day)	Fourth quartile (≥788 μg/day)	
	*n* = 24 875	*n* = 6198	*n* = 6238	*n* = 6213	*n* = 6226	
**DQX analysis entry age [years, mean (SD)]**	62.58 (5.35)	61.93 (5.29)	63.02 (5.32)	62.94 (5.29)	62.45 (5.40)	<0.001
**Study arm (%)**						
Intervention	24 875 (100)	6198 (100)	6238 (100)	6213 (100)	6226 (100)	
**Race/ethnicity (%)**						<0.001
Non-Hispanic white	22 631 (90.98)	5805 (93.66)	5865 (94.02)	5807 (93.47)	5154 (82.78)	
Non-Hispanic black	1057 (4.25)	302 (4.87)	275 (4.41)	268 (4.31)	212 (3.41)	
Asian	736 (2.96)	5 (0.08)	16 (0.26)	30 (0.48)	685 (11)	
Other	446 (1.79)	85 (1.37)	81 (1.30)	105 (1.69)	175 (2.81)	
Missing	5 (0.02)	1 (0.02)	1 (0.02)	3 (0.05)	0 (0)	
**BMI [kg/m** ^**2**^ **, mean (SD)]**	26.95 (5.40)	27.28 (5.44)	27.15 (5.43)	26.96 (5.37)	26.43 (5.31)	<0.001
**BMI category (%)**						<0.001
<18.5	273 (1.10)	47 (0.76)	76 (1.22)	57 (0.92)	93 (1.49)	
18.5–25	10 078 (40.51)	2408 (38.85)	2435 (39.03)	2477 (39.87)	2758 (44.30)	
25–30	8532 (34.30)	2123 (34.25)	2146 (34.40)	2219 (35.72)	2044 (32.83)	
>30	5765 (23.18)	1558 (25.14)	1538 (24.66)	1395 (22.45)	1274 (20.46)	
Missing	227 (0.91)	62 (1)	43 (0.69)	65 (1.05)	57 (0.92)	
**Education (%)**						<0.001
High school or less	8319 (33.44)	2434 (39.27)	2360 (37.83)	1948 (31.35)	1577 (25.33)	
Post-high school training or some college	8899 (35.77)	2253 (36.35)	2152 (34.50)	2315 (37.26)	2179 (35)	
College graduate or post-graduate	7639 (30.71)	1506 (24.30)	1720 (27.57)	1946 (31.32)	2467 (39.62)	
Missing	18 (0.07)	5 (0.08)	6 (0.10)	4 (0.06)	3 (0.05)	
**Smoking status (%)**						<0.001
Never smoker	14 040 (56.44)	3307 (53.36)	3500 (56.11)	3598 (57.91)	3635 (58.38)	
Former smoker	2264 (9.10)	676 (10.91)	613 (9.83)	520 (8.37)	455 (7.31)	
Current smoker	8571 (34.46)	2215 (35.74)	2125 (34.07)	2095 (33.72)	2136 (34.31)	
**Family history of ovarian cancer (%)**						0.739
Yes	23 458 (94.30)	5855 (94.47)	5871 (94.12)	5847 (94.11)	5885 (94.52)	
No	973 (3.91)	244 (3.94)	251 (4.02)	252 (4.06)	226 (3.63)	
Missing	444 (1.78)	99 (1.60)	116 (1.86)	114 (1.83)	115 (1.85)	
**Alcohol use (%)**						<0.001
No	6442 (25.90)	1896 (30.59)	1459 (23.39)	1428 (22.98)	1659 (26.65)	
Yes	18 433 (74.10)	4302 (69.41)	4779 (76.61)	4785 (77.02)	4567 (73.35)	
**Parity (%)**						<0.001
No live births	2201 (8.85)	490 (7.91)	511 (8.19)	536 (8.63)	664 (10.66)	
≥1 live birth	22 635 (90.99)	5697 (91.92)	5715 (91.62)	5670 (91.26)	5553 (89.19)	
Missing	39 (0.16)	11 (0.18)	12 (0.19)	7 (0.11)	9 (0.14)	
**Years of oral contraceptive use (%)**						<0.001
Not applicable	11 400 (45.83)	2666 (43.01)	2926 (46.91)	2956 (47.58)	2852 (45.81)	
≤5	8147 (32.75)	2096 (33.82)	2006 (32.16)	1971 (31.72)	2074 (33.31)	
>5	5285 (21.25)	1428 (23.04)	1296 (20.78)	1277 (20.55)	1284 (20.62)	
Missing	43 (0.17)	8 (0.13)	10 (0.16)	9 (0.14)	16 (0.26)	
**Years of female hormones use (%)**						<0.001
Not applicable	8680 (34.89)	2198 (35.46)	2324 (37.26)	2129 (34.27)	2029 (32.59)	
≤5	7881 (31.68)	1923 (31.03)	1907 (30.57)	1963 (31.60)	2088 (33.54)	
>5	8143 (32.74)	2044 (32.98)	1971 (31.60)	2074 (33.38)	2054 (32.99)	
Missing	171 (0.69)	33 (0.53)	36 (0.58)	47 (0.76)	55 (0.88)	
**Total energy intake [kcal/day, median (IQR)]**	1658.97 (1318.05, 2085.91)	1421.00 (1,130.85, 1760.24)	1631.35 (1301.58, 1992.99)	1758.69 (1415.55, 2178.50)	1862.93 (1492.47, 2,325.67)	<0.001
**Ovarian cancer (%)**						0.624
No	24 646 (99.08)	6149.00 (99.21)	6175.00 (98.99)	6154.00 (99.05)	6168.00 (99.07)	
Yes	229 (0.92)	49.00 (0.79)	63.00 (1.01)	59.00 (0.95)	58.00 (0.93)	

The correlation between the PEs intake and risk for developing ovarian cancer was then analyzed after being modified by potential variables, as detailed in Table 3. For the total PEs intake, women with the *Q*_4_ of total PEs had a decreased risk as compared with the *Q*_1_ in the DHQ group, as evidenced by the multi-adjusted HR (95% CI) of 0.69 (0.50–0.95) for DHQ. However, no significant linear trends were observed for the DHQ group (*P* for trend = 0.066). Notably, for the DHQ group, individuals who consumed the *Q*_4_ of isoflavones had a significant protective effect for ovarian cancer risk (HR_*Q*4vs*Q*1_: 0.68, 95% CI: 0.50–0.94; *P* for trend = 0.032). However, no such significant associations were observed for the DQX group. Another subtype of PEs, named coumestrol, have also no association with risk of ovarian cancer in both DQX (HR_*Q*4vs*Q*1_: 0.99, 95% CI: 0.67–1.45) and DHQ groups (HR_*Q*4vs*Q*1_: 0.89, 95% CI: 0.63–1.25), with the linear trends were not significant (*P* = 0.744 for DQX and *P* = 0.571 for DHQ). The restricted cubic spline models were illustrated in “Supplementary Figures S1–S3 is available at *Carcinogenesis* Online”, a reduced but not statistically significant ovarian cancer risk was observed as dietary total PEs and isoflavones intakes increased. No evidence of significant non-linearity was observed.

**Table 3.** HRs (95% CI) for ovarian cancer according to quintiles (*Q*) of intakes of total and individual phytoestrogens in the PLCO, 1993–2009

Phytoestrogen	DQX		DHQ	
	Unadjusted-model HR (95% CI)	Multi-adjusted-model^a^ HR (95% CI)	Unadjusted-model HR (95% CI)	Multi-adjusted-model^a^ HR (95% CI)
**Total phytoestrogens (μg/day)**				
*Q*_1_	Reference	Reference	Reference	Reference
*Q*_2_	1.13 (0.78, 1.64)	1.10 (0.76, 1.60)	0.77 (0.56, 1.06)	0.77 (0.56, 1.05)
*Q*_3_	1.06 (0.72, 1.55)	1.02 (0.70, 1.49)	0.91 (0.67, 1.23)	0.89 (0.65, 1.20)
*Q*_4_	1.06 (0.72, 1.56)	1.03 (0.70, 1.52)	**0.71 (0.51, 0.98)** ^*^	**0.69 (0.50, 0.95)** ^*^
*P* for linear trend	0.881	0.985	0.097	0.066
**Total isoflavones (μg/day)**				
*Q*_1_	Reference	Reference	Reference	Reference
*Q*_2_	1.10 (0.76, 1.60)	1.08 (0.74, 1.58)	0.82 (0.60, 1.11)	0.80 (0.59, 1.10)
*Q*_3_	0.98 (0.67, 1.44)	0.95 (0.64, 1.39)	0.85 (0.62, 1.15)	0.84 (0.62, 1.14)
*Q*_4_	1.11 (0.76, 1.61)	1.08 (0.74, 1.57)	**0.71 (0.51, 0.97)** ^*^	**0.68 (0.50, 0.94)** ^*^
*P* for linear trend	0.765	0.878	**0.047**	**0.032**
**Coumestrol (μg/day)**				
*Q*_1_	Reference	Reference	Reference	Reference
*Q*_2_	1.07 (0.73, 1.56)	1.06 (0.72, 1.54)	1.12 (0.82, 1.54)	1.11 (0.81, 1.52)
*Q*_3_	1.32 (0.92, 1.89)	1.30 (0.90, 1.86)	1.17 (0.85, 1.60)	1.14 (0.83, 1.56)
*Q*_4_	1.00 (0.68, 1.47)	0.99 (0.67, 1.45)	0.91 (0.65, 1.27)	0.89 (0.63, 1.25)
*P* for linear trend	0.696	0.744	0.667	0.571

^a^Adjusted for DHQ/DQX analysis entry age, BMI category, family history of ovarian cancer, years of oral contraceptive use and years of female hormones use. Bold values indicate a statistical significance.

^*^ *P* < 0.05.

We then performed stratified analyses based on BMI (<25 and ≥25 kg/m^2^) and age (<65and ≥65 years) in DHQ (Tables 4 and 5). Reverse associations were obtained between total PEs and the risk of ovarian cancer in BMI ≥ 25 kg/m^2^ (HR_*Q*4vs*Q*1_: 0.59, 95% CI: 0.37–0.95; *P* for trend = 0.085) and age ≥ 65 years (HR_*Q*4vs*Q*1_: 0.51, 95% CI: 0.33–0.79; *P* for trend = 0.008). Similar associations were investigated on total isoflavones (BMI ≥ 25 kg/m^2^: HR_*Q*4vs*Q*1_: 0.52, 95% CI: 0.33–0.84; *P* for trend = 0.012; age ≥ 65 years: HR_*Q*4vs*Q*1_: 0.49, 95% CI: 0.31–0.77; *P* for trend = 0.006). No significant interactions were found between the BMI and age and PE intake.

**Table 4.** Stratified analyses on the associations between phytoestrogens and ovarian cancer in the PLCO, 1993–2009

Phytoestrogen	BMI category		*P* for interaction
	<25 kg/m^2^	≥25 kg/m^2^	
	HR (95% CI)^a^	HR (95% CI)^a^	
**Total phytoestrogens (μg/day)**			0.656
*Q*_1_	Reference	Reference	
*Q*_2_	0.72 (0.45, 1.16)	0.82 (0.54, 1.27)	
*Q*_3_	0.88 (0.56, 1.38)	1.00 (0.67, 1.50)	
*Q*_4_	0.75 (0.47, 1.20)	**0.59 (0.37, 0.95)** ^*^	
*P* for linear trend	0.367	0.085	
**Total isoflavones (μg/day)**			0.572
*Q*_1_	Reference	Reference	
*Q*_2_	0.74 (0.46, 1.18)	0.81 (0.54, 1.23)	
*Q*_*3*_	0.85 (0.54, 1.33)	0.84 (0.55, 1.27)	
*Q*_4_	0.73 (0.46, 1.16)	**0.52 (0.33, 0.84)** ^**^	
*P* for linear trend	0.262	**0.012**	
**Coumestrol (μg/day)**			0.749
*Q*_1_	Reference	Reference	
*Q*_2_	1.49 (0.93, 2.41)	1.10 (0.72, 1.68)	
*Q*_3_	1.55 (0.97, 2.48)	1.06 (0.69, 1.63)	
*Q*_4_	1.18 (0.71, 1.94)	0.76 (0.47, 1.23)	
*P* for linear trend	0.443	0.286	

^a^Adjusted for DHQ analysis entry age, family history of ovarian cancer, years of oral contraceptive use and years of female hormones use. Bold values indicate a statistical significance.

^*^ *P* < 0.05.

^**^ *P* < 0.05.

**Table 5.** Stratified analyses on the associations between phytoestrogens and ovarian cancer in the PLCO, 1993–2009

Phytoestrogen	Age category		*P* for interaction
	<65 y	≥65 y	
	HR (95% CI)^a^	HR (95% CI)^a^	
**Total phytoestrogens (μg/day)**			0.138
*Q*_1_	Reference	Reference	
*Q*_2_	1.17 (0.72, 1.91)	0.64 (0.43, 0.97)	
*Q*_3_	1.04 (0.63, 1.72)	0.78 (0.53, 1.15)	
*Q*_4_	1.07 (0.65, 1.77)	**0.51 (0.33, 0.79)** ^**^	
*P* for linear trend	0.914	**0.008**	
**Total isoflavones (μg/day)**			0.302
*Q*_1_	Reference	Reference	
*Q*_2_	0.88 (0.54, 1.43)	0.72 (0.48, 1.07)	
*Q*_3_	0.82 (0.50, 1.35)	0.84 (0.57, 1.24)	
*Q*_4_	0.94 (0.59, 1.52)	**0.49 (0.31, 0.77)** ^**^	
*P* for linear trend	0.756	**0.006**	
**Coumestrol (μg/day)**			0.374
*Q*_1_	Reference	Reference	
*Q*_2_	1.56 (0.94, 2.59)	0.93 (0.62, 1.40)	
*Q*_3_	1.51 (0.93, 2.43)	0.91 (0.60, 1.37)	
*Q*_4_	1.12 (0.66, 1.89)	0.78 (0.50, 1.20)	
*P* for linear trend	0.607	0.268	

^a^Adjusted for BMI category, family history of ovarian cancer, years of oral contraceptive use and years of female hormones use. Bold values indicate a statistical significance.

^**^ *P* < 0.01.

## Discussion

In this study, we observed a significant association between total PE intake and ovarian cancer risk. Especially, the analysis stratified by total isoflavones intake revealed a significantly reduced risk of ovarian cancer, supporting a protective effect of isoflavones with a linear trend.

As one of the primary female sex steroid hormones, estrogens play critical roles in regulating cell growth, differentiation and maintaining the function of female reproductive tissues, especially for the majority of post-menopausal women in our study (6). However, numerous evidences strongly suggest that excess exposure to estrogen is implicated in ovarian carcinogenesis (7). The PEs are mainly isolated from soybean, which is popular in East Asia countries including China. Multiple studies have found that a higher dietary intake of PEs could decrease the incidence of several cancers such as lung cancer and even hormone-dependent breast, endometrial, prostate and thyroid cancers (15–17). In the current study, although the PEs display weak estrogen-like properties, our study revealed that the daily intake of total PEs was linked with reduced ovarian cancer risk. This finding was different from a previous population-based cohort study with 47 140 Swedish women by Hedelin *et al*. (18), which demonstrated no statistically significant association between specific food items rich in PEs and overall ovarian cancer risk. Another case-control study performed by Bandera *et al*. (19) also reported the intake of PEs had little impact on ovarian cancer risk, though the point estimates were below one.

The PEs are usually divided into three major classes including isoflavones, lignans and coumestrol, among which isoflavones are the most investigated (8,20). Our analysis demonstrated that the highest intake of dietary isoflavones significantly decreased the risk of ovarian cancer (HR: 0.68, 95% CI: 0.49, 0.94; *P* for trend = 0.030), which was similar to the results from the previous meta-analysis (21). In the meta-analysis, Qu *et al*. (21) found that isoflavones were confirmed as the only subtype of PEs to be linked with decreased risk of ovarian cancer (RR: 0.63, 95% CI: 0.46, 0.86; *P* = 0.008). In general, the dietary isoflavones can be bio-transformed to equol based on the decomposition of gut bacteria, which shows a high binding affinity to estrogen receptor (ER) (22,23). In this scenario, the equol could work by competing with 17β-estradiol to bind to the ERs, accounting for the potential inhibitory effect in ovarian carcinogenesis (22). To date, accumulated experimental studies have provided the evidence for the anticancer effects of isoflavones in ovarian cancer (10–13). For example, researchers have found that the PEs and their synthetic analogues could control ovarian and breast cancers by downregulating the recognized oncoprotein CYP1B1 during the late 1990s (10). Sahin *et al*. (11) demonstrated that intake of genistein, the major type of isoflavone in soybean, effectively reduced the occurrence of ovarian tumors in the laying hen model in which spontaneous ovarian cancer develops at high incidence rates. Another experimental study by Kim *et al*. (12) reported that genistein could inhibit epithelial–mesenchymal transition (EMT) and migration of BG-1 ovarian cancer cells via competing to bind ERs and downregulating the tumor growth factor β (TGF-β) signaling pathway. However, it should not be ignored that there are also studies reporting the potential tumorigenic effects of PEs. Endocrine disruptors (EDCs) are defined as chemicals that damage the homeostasis of the endocrine system in the human body. Notably, the PEs are also regarded as one type of EDCs and can be found in our diet such as red wine, which can lead to infertility, premature puberty and even cause ovarian cancer (24). Moreover, Wang *et al*. (25) conducted a mechanistic study and showed that genistein could promote the proliferation of ovarian cancer OVCAR-5 cells by increasing the expression of cyclin D1 and CDK4. Therefore, it is difficult to recommend isoflavones as a treatment for ovarian cancer prevention. Further studies into the molecular mechanism of isoflavones in promoting or inhibiting ovarian cancer in more detail are recommended.

Then the stratified analyses based on BMI and age in DHQ were performed. A reduced ovarian cancer risk was observed in both total PEs and total isoflavones intakes in women whose age ≥ 65 years and in women with a BMI ≥ 25 kg/m^2^. Indeed, older age and obesity are two important risk factors for ovarian cancer development and progression (26,27). Particularly, epithelial ovarian cancer represents the most common histology type of all ovarian cancer subtypes, and the median age of EOC at diagnosis is 63 years (28). Our results suggested that both total PEs and isoflavones intakes may provide a protective role in ovarian carcinogenesis in obese or older women. However, no significant interactions were found between BMI and age and PE intake. This may be due to the inadequacy of sample sizes or limitations of the sample source region. Further analysis with larger sample sizes is needed to elucidate our results.

The unique strengths of our study are based on a standardized assessment of PE intake and detailed information on various confounders. Furthermore, due to the prospective design, multiple screening centers, and long follow-up period of this study, the potential selection bias could be minimized and the results are less likely from reverse causation. Additionally, the interaction test is performed and the *P* value is not significant based on the hierarchical analysis of different variables, indicating the consistency and reliability of the results. Despite its strengths, the results of the present study should be interpreted within the context of several limitations. First, the exposure was acquired from self-reported dietary data, which leads to a possibility of misclassification and causes underestimated associations between PE intake and ovarian cancer risk. Second, lignan is one of the major components of PEs but such dietary data are not available in the PLCO, potentially underestimating the effect of total PEs intake on ovarian cancer risk. Third, the differences in the absorption or metabolism of PEs, caused by individual heterogeneities in dietary habits, food matrix, and gut microbia, may account for the discrepancies in results (29).

In conclusion, increased dietary intake of total PEs especially isoflavones could reduce the risk of ovarian cancer in this trial. Further studies are also needed to replicate the results and exploit the underlying mechanism of the protective effects.

## Supplementary material

Supplementary data are available at *Carcinogenesis* online.

bgae015_suppl_Supplementary_Figures_S1-S3

## Data Availability

Data described in the manuscript will be shared on reasonable request pending approval by the PLCO.

## Abbreviations

CI: confidence interval
DHQ: diet history questionnaire
DQX: dietary questionnaire
EOC: epithelial ovarian cancer
HR: hazard ratios
PE: phytoestrogens
PLCO: prostate, lung, colorectal and ovarian
