# Supplementary material for: Dietary phytoestrogen intake and ovarian cancer risk: a prospective study in the prostate, lung, colorectal and ovarian (PLCO) cohort
Source: Carcinogenesis. 2024 Feb 20;45(6):378–86. doi: 10.1093/carcin/bgae015 (PMC11164104; doi:10.1093/carcin/bgae015)
Supplement: bgae015_suppl_Supplementary_Figures_S1-S3 [file bgae015_suppl_supplementary_figures_s1-s3.docx]

**Supplementary Materials**

**Dietary phytoestrogen intake and ovarian cancer risk: a prospective study in the Prostate, Lung, Colorectal and Ovarian (PLCO) cohort**

Yizuo Song^1,2†^, Huijun Huang^3†^, Mingmin Jin^3^, Binwei Cheng^3^, Shanshan Wang^3^, Xinjun Yang^3*^, Xiaoli Hu^1,2*^

*^1^Department of Obstetrics and Gynecology, the First Affiliated Hospital of Wenzhou Medical University, Wenzhou 325000, Zhejiang, China*

*^2^Zhejiang Provincial Clinical Research Center for Obstetrics and Gynecology, the First Affiliated Hospital of Wenzhou Medical University, Wenzhou 325000, Zhejiang, China*

*^3^Department of Epidemiology and Health Statistics, School of Public Health and Management, Wenzhou Medical University, Wenzhou, Zhejiang, China*

^†^These authors contributed equally to this work.


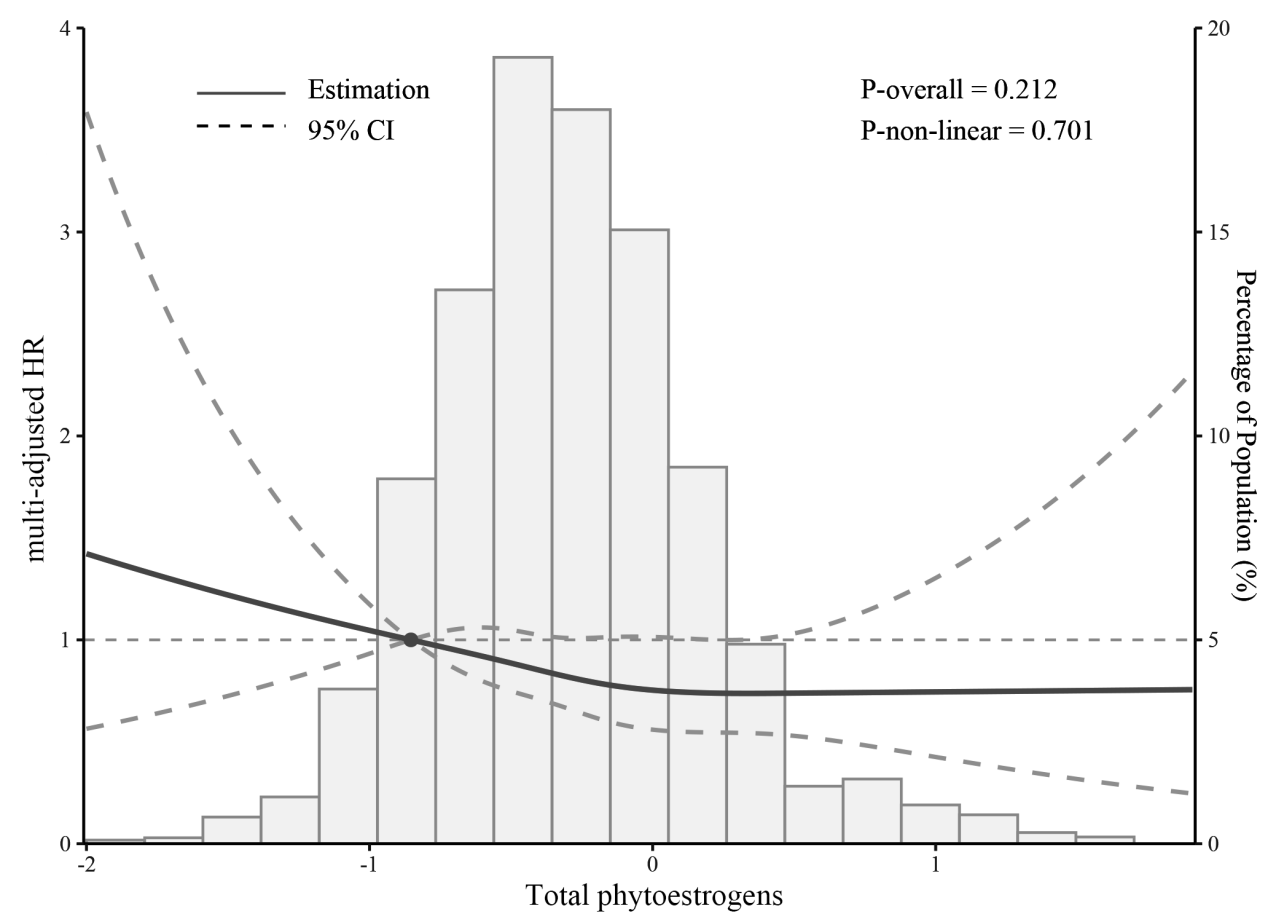


**Figure S1.** Dose-response using restricted cubic spline model for the association between total phytoestrogens intake and ovarian cancer risk in PLCO cohort. Solid line represents point estimates and dashed lines represent 95% CIs. Multivariable risk estimate was calculated by restricted cubic spline regression (using three knots at 5th, 35th, 65th, and 95th percentiles) adjusting for DHQ analysis entry age, BMI category, family history of ovarian cancer, years of oral contraceptive use, years of female hormones use. Log transformation was used because of the positive skew of total phytoestrogens intake. Individuals with zero total phytoestrogens intake were excluded (n=2).


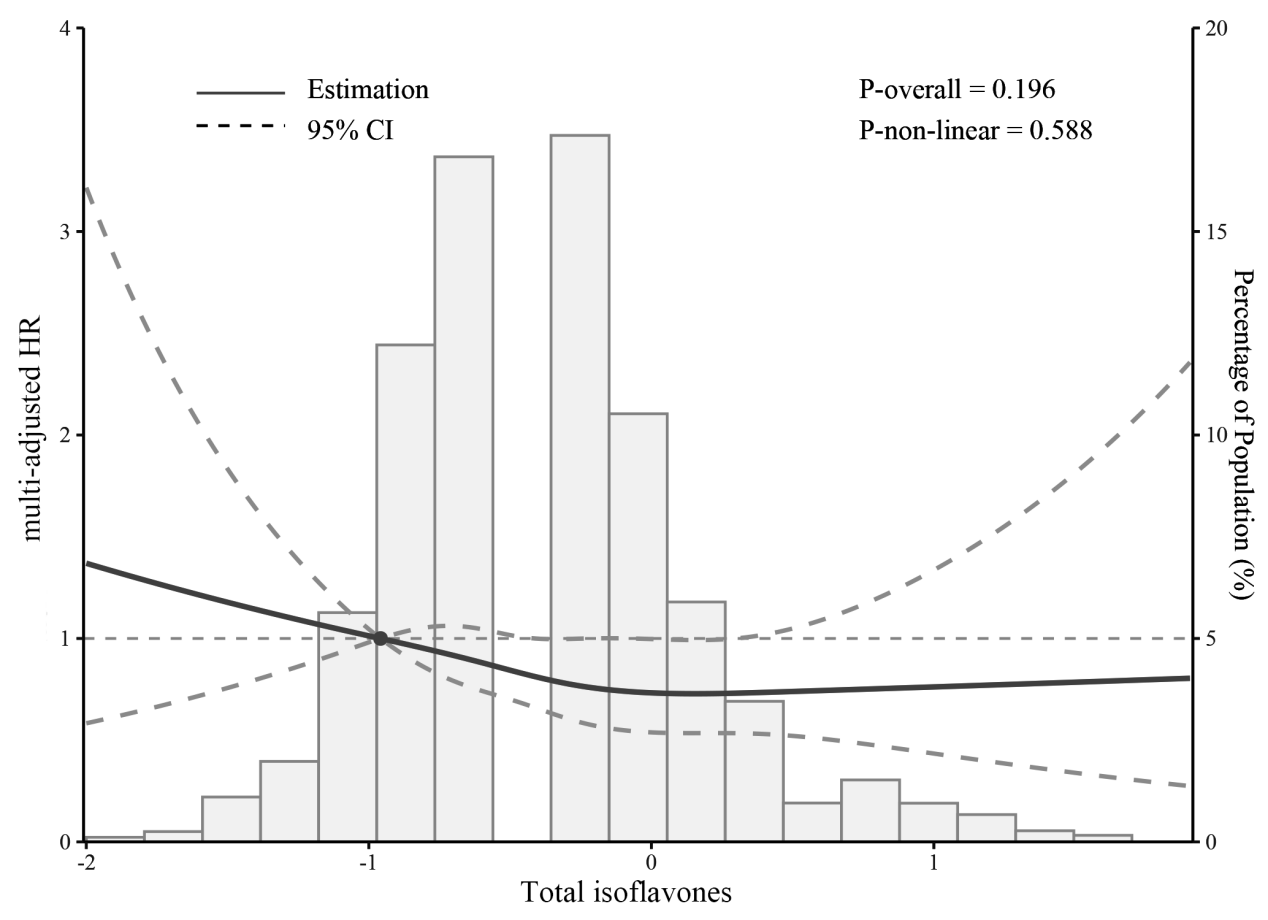


**Figure S2.** Dose-response using restricted cubic spline model for the association between total isoflavones intake and ovarian cancer risk in PLCO cohort. Solid line represents point estimates and dashed lines represent 95% CIs. Multivariable risk estimate was calculated by restricted cubic spline regression (using three knots at 5th, 35th, 65th, and 95th percentiles) adjusting for DHQ analysis entry age, BMI category, family history of ovarian cancer, years of oral contraceptive use, years of female hormones use. Log transformation was used because of the positive skew of total isoflavones intake. Individuals with zero total isoflavones intake were excluded (n=3).


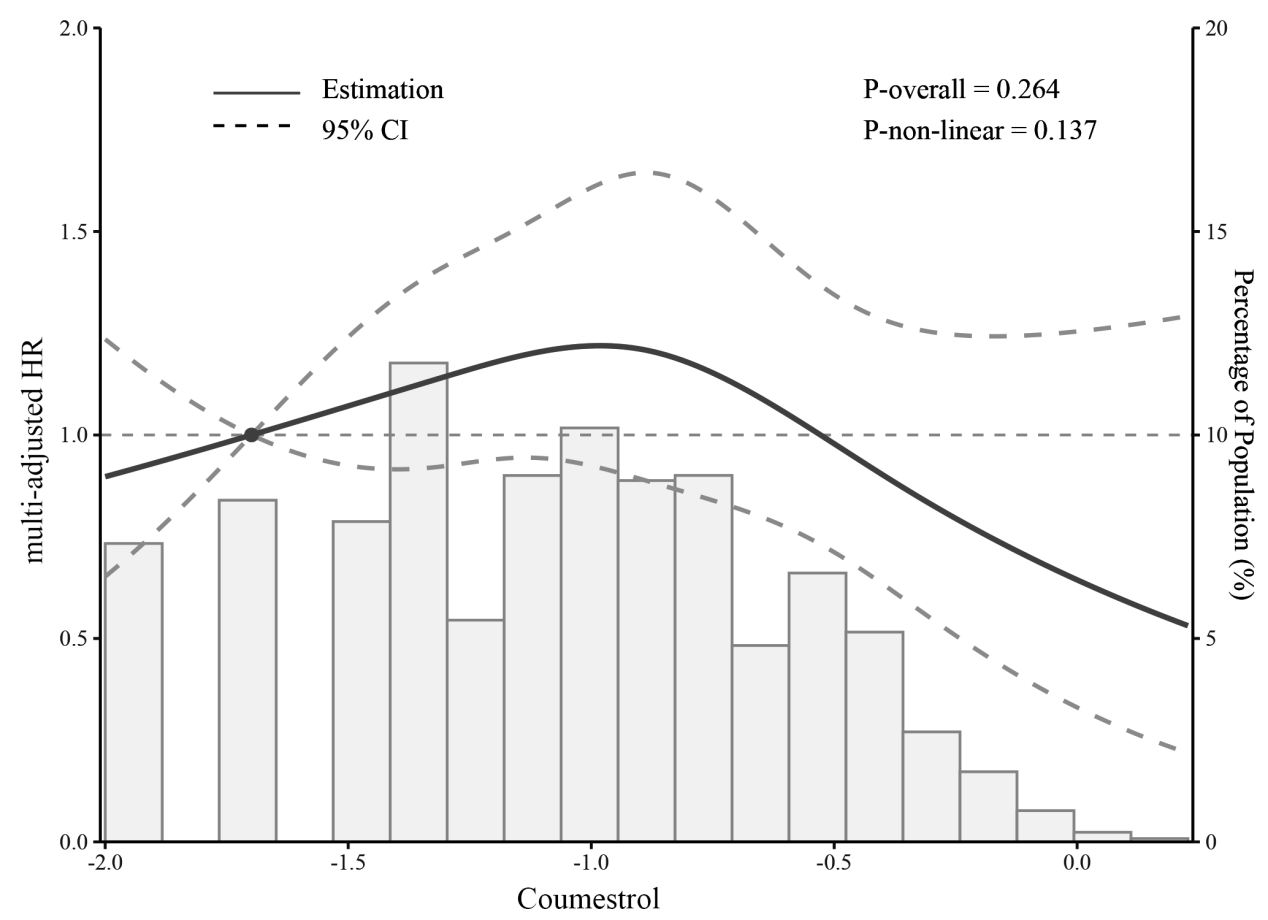


**Figure S3.** Dose-response using restricted cubic spline model for the association between coumestrol intake and ovarian cancer risk in PLCO cohort. Solid line represents point estimates and dashed lines represent 95% CIs. Multivariable risk estimate was calculated by restricted cubic spline regression (using three knots at 5th, 35th, 65th, and 95th percentiles) adjusting for DHQ analysis entry age, BMI category, family history of ovarian cancer, years of oral contraceptive use, years of female hormones use. Log transformation was used because of the positive skew of total coumestrol. Individuals with zero coumestrol intake were excluded (n=1029).
